# Interaction of fatty acid quality indices and genes related to lipid homeostasis on mental health among overweight and obese women

**DOI:** 10.1038/s41598-023-35810-4

**Published:** 2023-06-13

**Authors:** Niloufar Rasaei, Alireza Khadem, Lilit Sardari masihi, Khadijeh Mirzaei

**Affiliations:** 1 Department of Community Nutrition, School of Nutritional Sciences and Dietetics, Tehran University of Medical Sciences (TUMS), P.O. Box:14155-6117, Tehran, Iran; 2 Department of Nutrition, Science and Research Branch, Islamic Azad University, Tehran, Iran; 3 Food Microbiology Research Center, Tehran University of Medical Sciences, Tehran, Iran

**Keywords:** Genetics research, Psychiatric disorders

## Abstract

The aim of this study is to investigate the interaction of fatty acid quality indices and genes related to lipid homeostasis on mental health among overweight and obese women. This cross-sectional study included 279 overweight and obese women for N6/N3 ratio and 378 overweight and obese women for CSI aged 18–58 years. Mental health were evaluated using Depression Anxiety Stress Scales (DASS-21). The anthropometric indices, biochemical parameters, body composition and dietary fat quality were measured. MC4R (rs17782313) and Caveolin-1 (CAV-1) (rs3807992) were genotyped by polymerase chain reaction-restriction fragment length polymorphism (PCR–RFLP) technique. The results of the study showed that after adjusting for age, energy intake, thyroid disease, physical activity, and BMI, a positive interaction between TC genotype of MC4R and CSI on depression (β = 0.39, CI = 0.12, 0.66, P = 0.004), and DASS-21 (β = 0.074, CI = 0.04, 1.44, P = 0.036). Also, there were a marginal significant interactions between AG genotype of CAV-1 and N6/N3 ratio on depression in adjustment model1 (β = 16.83, CI = − 0.19, 33.85, P = 0.053). Our findings showed that increasing adherence to fatty acid quality indices by considering genes related to lipid homeostasis was related to increasing depression in our population.

## Introduction

Mental health and its contributing factors have been increasingly studied by health researchers in the post-coronavirus era. The importance of accurate knowledge of the factors that prevent the development of depression, stress, and anxiety has become more obvious. Research in this field is important because it can reduce the burden of long-term treatment costs for these patients on the health system^1^. Research published before the spread of the coronavirus reported that the prevalence of anxiety and depression was 7.3% and 4.7% of the world’s population, respectively. However, this research did not take into account national quarantines’ outcomes on mental health. Therefore, it is important to pay attention to all aspects affecting mental health^2^. Obese women are twice as likely to be diagnosed with depression and anxiety disorders compared to obese men^3^. Women who are overweight also have a higher risk of depression than those with normal weight^4^. In recent decades, societies have become industrialized and people have migrated to cities, causing significant changes in people’s lifestyles and the environment. However, our genetic changes have not adapted to these changes and remain an evolutionary mismatch^5^. Unhealthy eating is one of the most obvious consequences of modern life and is the cause of many non-communicable and mental diseases^6^. The importance of dietary factors is such that according to research in 2017, nutritional risks were placed at level 2 for risk factors of mortality^7^. Due to changes in lifestyle and diet over the past centuries, the percentage of intake of many fatty acids has changed significantly. According to research, in the United States of America, the daily intake of omega-6 linoleic acid has increased from 3% of total energy intake to more than 7%^8^. According to meta-analysis conducted by Zhang and et al. the amount of omega-3 intake has a significant effect on reducing stress and depression^9^. Changes in the ratio of omega-6 to omega-3 can affect mental health. Although many studies support the theory of PUFA consumption, especially omega-3, as reducing depression, clinical trials have also been conducted that have not observed significant effect^10,11^.

Based on the previous statements, it can be assumed that genes affecting lipid homeostasis may also have indirect effects on people’s mental health. The melanocortin-4 receptor (MC4R) gene is involved in regulating energy homeostasis and has been linked to obesity and metabolic consequences^12^. A study published in 2011 found that a variant near the MC4R gene regulates triglyceride levels^13^. Also Caveolin-1 (Cav-1) controls the metabolism of lipid droplets in endothelial cells by stimulating autocrine prostacyclin and cAMP-mediated lipolysis. Lipid droplets (LD) are organelles that are involved in intracellular lipid metabolism in almost all eukaryotic cells, and their dynamics are tightly regulated by proteins associated with them^14^. Given the importance of mental health to society and the possibility of indirect effects of these genes on mental health, further research is needed to investigate these connections.

## Methods

### Study population

This study was conducted in Tehran, Iran and included 378 women who were overweight or obese. The subjects were between the ages of 18–68 and had a body mass index (BMI) of 25–40 kg/m^2^. Women who were pregnant or at menopause, lactating, smoking, dieting, taking weight loss supplements, antipsychotics, antihypertensives, or lowering glucose or lipid levels were excluded from the study. Women with malignancies, depression, diabetes, liver disease, kidney disease, cardiovascular disease or other chronic or acute diseases were also excluded. The study was approved by the Ethics Committee of the Tehran University of Medical Sciences (TUMS) and received ethical approval (assigned number: IR.TUMS.MEDICINE.REC.1401.504). Overweight and obese women were recruited from all regional health centers (n = 20) in western and central Tehran using a community-based multistage simple random sampling method. Participants were chosen at random from Tehran health centers if they met the criteria for inclusion in the study. The participants’ variables were classified according to N6/N3 and CSI quartiles, with Q1 and Q4 being the lowest and highest quartiles of N6/N3 and CSI, respectively.

### Sociodemographic and blood pressure measurements

The study used a standard sociodemographic questionnaire to assess employment, education level, marital and economic status, and supplement intake. The participants’ weight, BMI, and body fat percent (BF%) were analyzed using a bioelectrical impedance analyzer (BIA) (InBody 770 scanner, Seoul, Korea)^15^. Height was measured using a non-elastic tape and waist circumference (WC) and the hip circumference (HC) was measured using an elastic tape. The waist-to-hip ratio (WHR) is calculated by dividing the WC by the hip circumference (HC). Blood pressure was measured twice with an appropriate cuff after 5 min of rest and the mean value was recorded. Physical activity levels of the participants were gathered using the short form of a reliable and validated international physical activity questionnaire (IPAQ)^16^. Metabolic equivalent hours per week (METs-h/week) were measured for each subject during the previous week.

### Biochemical variables

For venous blood sampling, the patients came to the center after fasting for 10 h, and after collecting and centrifuging the serum, it was kept at a temperature of –80 °C. In the biochemistry laboratory located in the University of Nutrition and Dietetics, standard methods were used to evaluate the collected samples. The glycerol-3-phosphate oxidase-phenol 4-aminoantipyrine peroxidase (GPOPAP) enzymatic method was used to measure TG and TC. And the amount of HDL was also measured after the precipitation of lipoproteins containing apo B Also, LDL was measured by direct enzymatic method, and to measure insulin from the Access Chemiluminescent Immunoassay method. Using the homeostatic model evaluation formula (HOMA), the index of insulin resistance was evaluated $\frac{\mathrm{fasting plasma glucose }\times \mathrm{fasting serum insulin}}{405}$.

### Mental health assessment

The mental health status of participants was evaluated using the DASS-21 questionnaire, which is a short version of a 42-item self-report instrument designed to measure three related negative emotional states: depression, anxiety, and tension/stress. It consists of three self-report scales that are used to measure the emotional states of depression, anxiety, and stress. Each of the three DASS-21 scales contains 7 items, divided into subscales with similar content. The individual is required to indicate the presence of a symptom over the previous week^17^. To determine the final score, the DASS-21 scores were multiplied by two and divided into three categories as thresholds for depression (≥ 10), anxiety (≥ 8), and stress (≥ 15).

### Dietary intake assessment and fatty acid quality indices calculation

We assessed the food intake of all participants over the past year using a validated standard semi-quantitative 147-item food frequency questionnaire (FFQ)^18^. A nutritionist completed this tool and subjects reported their frequency of food items on a daily, weekly, monthly, or yearly basis. Nutrient compositions and energy intakes of each food item were estimated using the NUTRITIONIST 4 food analyzer (First Data Bank, San Bruno, CA). The saturated fat cholesterol index (CSI) and omega-6/omega-3 essential fatty acid ratio (N-6/N-3) indices were considered as indices of dietary fat quality. Accordingly, the CSI was calculated by dividing cholesterol by the concentration of saturated fat in foods derived from FFQs data^19^. A low CSI indicates a low level of cholesterol and/or saturated fat. Additionally, the ratio of N-6 to N-3 was calculated by dividing the N-6 to N-3 content of foods gathered from the FFQ^20,21^.

### Genotyping

By using saturated NACL solution, we first extracted DNA from the whole blood sample^22^. And with the instructions given, we evaluated the concentration of the extracted DNA with Nanodrop 8000 spectrophotometry and checked its integrity with the help of 1% agarose gel. MC4R genotypes were determined by TaqMan open array using single nucleotide polymorphism (SNP) method^23^. Using the primers introduced by Zlatohlavek et al.^24^. The sequence of MC4R (rs17782313) primers used are as follows: primers forward: 5-AAGTTCTACCTACCATGTTCTTGG-3; reverse: 5-TTCCCCCTGAAGCTTTTCTTGTCATTTTGAT-3. Three types of genotypes for this gene including CC, TT and CT were identified. The forward primer of CAV-1 (rs3807992) is 3′AGTATTGACCTGATTTGCCATG 5′ and the reverse primer is 5′ GTCTTCTGGAAAAAGCACATGA 3′. The fragments containing three genotypes including GG, AA, and GA were distinguished.

### Statistical analyses

A Kolmogorov–Smirnov test was performed to test the normality of the data. General characteristics of the participants were reported as mean ± standard deviation (SD) according to the fatty acid quality indices tertiles. A one-way analysis of variance (ANOVA) test was used to compare anthropometric indices, and biochemical variables between participants. Analysis of covariance (ANCOVA) was applied to remove confounding results. A generalized linear model (GLM) was used to assess the interactions between genes related to lipid homeostasis and N6/N3 and CSI on stress, anxiety and depression in crude and adjusted models. The results were adjusted for energy intake, age, BMI, PA and thyroid disease. Statistical analyses were done using SPSS version 23.0 (SPSS, Chicago, IL, USA). P-values ​​less than 0.05 were considered statistically significant and P-values ​​less than 0.07 were considered marginally significant.

### Ethics approval and consent to participate

Ethics approval and consent to participate Ethics approval for the study protocol was confirmed by The Human Ethics Committee of Tehran University of Medical Sciences (TUMS) with the following identification: IR.TUMS.MEDICINE.REC.1401.504. All methods were carried out in accordance with relevant guidelines and regulations. Each participant was completely informed about the study protocol and provided a written and informed consent form before taking part in the study.

## Results

### Baseline characteristics of study population according to quartiles of CSI and N6/N3

The general characteristics of study participants, categorized according to quartiles of CSI and N6/N3, were presented in Tables 1 and 2. In the crude model, a significant mean differences among quartiles of the CSI in terms of SLM (P = 0.048), RMR (P = 0.030), and the significant mean difference in terms of WHR (P = 0.043), FFM (P = 0.041), SMM (P = 0.036), BMR (P = 0.042), and marginally significant for SLM (P = 0.057), were observed among quartiles of the N6/N3. After adjustment with confounders, including age, BMI, physical activity, and energy intake, the education (P = 0.026), BF% (P = 0.038), and FMI (P = 0.036) of participants among quartiles of the CSI became significant, and there was a significant mean differences in terms of HOMA index (P = 0.029) among quartiles of the N6/N3.

**Table 1:** General characteristics of study population according to quartiles of CSI (n = 378) in obese and overweight women.

Variables	CSI						
	Mean ± SD					P-value†	P-value _b_
	Q1 (n = 94)		Q2 (n = 95)	Q3 (n = 95)	Q4 (n = 94)		
Age (years)	38.55 ± 8.59		36.64 ± 9.90	36.76 ± 8.35	34.75 ± 9.62	**0.045**	**0.029**
PA (MET-min/week)	1128.17 ± 2569.97		1283.55 ± 2050.88	999.98 ± 926.49	1296.11 ± 2301.31	0.829	0.689
Anthropometric measurements							
Weight (kg)	79.71 ± 11.28		78.94 ± 9.83	82.06 ± 12.30	81.77 ± 11.60	0.159	0597
Height (cm)	160.52 ± 5.82		160.53 ± 5.86	162.25 ± 6.14	161.50 ± 5.17	0.114	0.813
WC (cm)	98.84 ± 9.52		97.67 ± 8.45	99.95 ± 10.13	100.27 ± 10.05	0.234	0.371
WHR (cm)	0.93 ± 0.49		0.92 ± 0.05	0.93 ± 0.05	0.94 ± 0.05	0441	0.428
BMI (kg/m^2^)	31.00 ± 3.94		30.60 ± 3.51	31.10 ± 3.78	31.34 ± 4.16	0.615	0.589
BF (%)	42.29 ± 5.20		41.62 ± 5.30	41.33 ± 5.56	42.93 ± 5.27	0.167	**0.038**
FFM (kg)	45.90 ± 5.30		45.71 ± 5.48	47.54 ± 5.97	46.27 ± 5.21	0.097	0.259
SMM (kg)	25.12 ± 3.15		25.18 ± 3.46	26.18 ± 3.56	25.37 ± 3.13	0.111	0.132
SLM (kg)	43.28 ± 5.02		42.72 ± 5.29	44.80 ± 5.62	43.62 ± 4.93	**0.048**	0.211
FMI	13.37 ± 3.30		12.89 ± 2.86	13.12 ± 2.93	13.64 ± 3.31	0.385	**0.036**
RMR	1530.04 ± 242.46		1532.76 ± 228.75	1638.30 ± 297.05	1574.63 ± 210.57	**0.030**	0.319
BMR	1361.45 ± 114.61		1357.60 ± 118.51	1397.01 ± 129.14	1380.12 ± 158.46	0.144	0.259
Biochemical variables							
TC (mg/dl)	182.90 ± 32.55	184.16 ± 33.55		186.15 ± 40.93	180.63 ± 36.54	0.884	0.793
TG (mg/dl)	124.39 ± 80.15	112.44 ± 59.53		133.79 ± 78.98	111.68 ± 51.82	0.258	0.319
HDL (mg/dl)	46.13 ± 10.76	48.70 ± 10.84		45.55 ± 11.53	46.02 ± 8.33	0.326	0.352
LDL (mg/dl)	94.68 ± 23.65	93.67 ± 23.31		93.23 ± 25.86	95.16 ± 23.15	0.939	0.282
Insulin (mIU/mL)	1.17 ± 0.22	1.21 ± 0.22		1.25 ± 0.23	1.21 ± 0.22	0.327	0.380
HOMA index	3.32 ± 1.43	3.27 ± 1.28		3.32 ± 1.10	3.50 ± 1.34	0.818	0.593
Education% (n)						0.169	**0.026**
Illiterate	50.0 (2)	50.0 (2)		0.0 (0)	0.0 (0)		
Primary education	42.2 (6)	7.7 (1)		23.1 (3)	23.1 (3)		
Intermediate Education	44.0 (11)	24.0 (6)		24.0 (6)	8.0 (2)		
High school education	25.0 (2)	37.5 (3)		12.5 (1)	25.0 (2)		
Diploma	23.1 (27)	26.5 (31)		28.2 (33)	22.2 (26)		
Postgraduate education	33.3 (9)	33.3 (9)		14.8 (4)	18.5 (5)		
Bachelor's degree and higher	19.8 (36)	23.1 (42)		26.4 (48)	30.8 (56)		
Marriage%(n)						0.194	0.694
Married	25.6 (69)	25.6 (69)		27.0 (73)	21.9 (59)		
Single	23.3 (21)	24.4 (22)		18.9 (17)	33.3 (30)		
Away from spouse more than 6 month	0.0 (0)	100.0 (2)		0.0 (0)	0.0 (0)		
Dead spouse	0.0 (0)	0.0 (0)		33.3 (1)	66.7 (2)		
Divorce	27.3 (3)	9.1 (1)		36.4 (4)	27.3 (3)		

BF%; body fat percentage; BMI: body mass index; BMR: basal metabolic rate; CSI: cholestrol to saturated fat index; FFM: fat free mass; FFMI: free fat mass index; FMI: fat mass index; HDL: high density lipoprotein; HOMA; homeostatic model assessment; Q: quartile; RMR: resting metabolic rate: SD: standard deviation; SLM: soft lean mass; SMM: skeletal muscle mass; TC: total cholesterol; TG: triglyceride; WC: waist circumference; WHR: waist-hip ratio.

^†^Calculated by analysis of variance (ANOVA).

b: Adjusted for age, BMI, physical activity, thyroid disease, and total energy intake.

Chi-square was used for categorical variables.

BMI consider as a collinear variable for anthropometric measurements and these variables adjusted for Age, physical activity, and total energy intake.

p < 0.05 was considered significant, and 0.05, 0.06, and 0.07 were considered marginally significant, and are bolded.

**Table 2:** General characteristics of study population according to quartiles of N6/N3 (n = 279) in obese and overweight women.

Variables†	N6/N3							
	Mean ± SD					P-value†	P-value _b_	
	Q1 (n = 69)		Q2 (n = 70)	Q3 (n = 70)	Q4 (n = 70)			
Age (years)	35.27 ± 8.41		35.95 ± 8.56	37.38 ± 7.79	37.30 ± 9.00	0.374	0.844	
PA (MET-min/week)	1571.96 ± 2774.24		910.92 ± 1124.72	1084.13 ± 1343.05	1148.57 ± 2433.89	0.338	0.675	
Anthropometric measurements								
Weight (kg)	81.50 ± 10.65		80.38 ± 11.57	80.95 ± 11.19	77.15 ± 9.74	0.081		0.671
Height (cm)	162.18 ± 5.48		161.45 ± 5.88	161.78 ± 5.59	159.88 ± 6.17	0.09		0.830
WC (cm)	99.14 ± 9.26		98.91 ± 9.75	99.50 ± 9.48	96.07 ± 8.52	0.114		0.287
WHR (cm)	0.93 ± 0.04		0.93 ± 0.05	0.94 ± 0.05	0.91 ± 0.04	**0.043**		0.107
BMI (kg/m^2^)	30.99 ± 4.03		30.92 ± 3.60	30.78 ± 3.57	30.22 ± 3.70	0.609		0.191
BF (%)	41.38 ± 6.33		41.14 ± 4.81	41.26 ± 4.89	41.28 ± 5.21	0.995		0.866
FFM (kg)	47.02 ± 4.75		47.34 ± 5.83	47.00 ± 5.62	45.01 ± 5.01	**0.041**		0.458
SMM (kg)	25.79 ± 2.80		26.07 ± 3.45	25.82 ± 3.36	24.62 ± 2.97	**0.036**		0.385
SLM (kg)	44.31 ± 4.47		44.62 ± 5.46	44.91 ± 5.54	42.42 ± 4.73	**0.057**		0.545
FMI (kg)	13.18 ± 3.28		12.86 ± 2.82	12.85 ± 2.69	12.74 ± 3.16	0.842		0.303
RMR	1610.51 ± 212.02		1563.17 ± 249.58	1584.01 ± 276.61	1520.74 ± 262.85	0.202		0.802
BMR	1385.57 ± 102.79		1392.75 ± 126.12	1385.47 ± 121.51	1342.30 ± 108.29	**0.042**		0.456
Biochemical variables								
TC (mg/dl)	178.67 ± 28.62	184.08 ± 37.04		185.81 ± 39.71	185.79 ± 36.88	0.682		0.773
TG (mg/dl)	121.74 ± 73.31	117.37 ± 66.52		119.63 ± 65.29	125.34 ± 74.94	0.935		0.728
HDL (mg/dl)	46.10 ± 9.35	46.79 ± 11.48		48.63 ± 11.50	45.32 ± 9.91	0.367		0.160
LDL (mg/dl)	91.49 ± 21.03	97.25 ± 22.90		93.34 ± 26.32	94.73 ± 24.79	0.624		0.219
Insulin (mIU/mL)	1.20 ± 0.23	1.25 ± 0.24		1.22 ± 0.23	1.17 ± 0.20	0.328		0.564
HOMA index	3.44 ± 1.33	3.05 ± 1.10		3.31 ± 1.33	3.53 ± 1.33	0.193		**0.029**
Education% (n)						0.973		0.872
Illiterate	0.0 (0)	33.3 (1)		33.3 (1)	33.3 (1)			
Primary education	23.1 (3)	30.8 (4)		30.8 (4)	30.8 (4)			
Intermediate Education	23.5 (4)	17.6 (3)		29.4 (5)	29.4 (5)			
High school education	28.6 (2)	28.6 (2)		28.6 (2)	14.3 (1)			
Diploma	27.2 (22)	28.4 (23)		22.2 (18)	22.2 (18)			
Postgraduate education	12.5 (3)	29.2 (7)		29.2 (7)	29.2 (7)			
Bachelor's degree and higher	25.8 (34)	22.7 (30)		24.2 (32)	27.3 (36)			
Marriage% (n)						0.337		0.410
Married	23.7 (51)	25.1 (54)		27.0 (58)	24.2 (52)			
Single	27.8 (15)	25.9 (14)		16.7 (9)	29.6 (16)			
Away from spouse more than 6 month	0.0 (0)	0.0 (0)		0.0 (0)	100.0 (1)			
Dead spouse	100.0 (2)	0.0 (0)		0.0 (0)	0.0 (0)			
Divorce	0.0 (0)	40.0 (2)		40.0 (2)	20.0 (1)			

BF%; body fat percentage; BMI: body mass index; BMR: basal metabolic rate; FFM: fat free mass; FFMI: free fat mass index; FMI: fat mass index; HDL: high density lipoprotein; HOMA; homeostatic model assessment; Q: quartile; RMR: resting metabolic rate: SD: standard deviation; SLM: soft lean mass; SMM: skeletal muscle mass; TC: total cholesterol; TG: triglyceride; WC: waist circumference; WHR: waist-hip ratio.

^†^Calculated by analysis of variance (ANOVA).

b: Adjusted for age, BMI, physical activity, thyroid disease, and total energy intake.

Chi-square was used for categorical variables.

BMI consider as a collinear variable for anthropometric measurements and these variables adjusted for Age, physical activity, and total energy intake.

p < 0.05 was considered significant, and 0.05, 0.06, and 0.07 were considered marginally significant, and are bolded.

### Dietary intake of participants according to quartiles of CSI and N6/N3

Dietary intakes of participant’s study population among quartiles of CSI and N6/N3 ratio were presented in Table 3,4. After adjustment with the energy intake, mean differences of grains, dairy, red meat, protein, carbohydrate, total fat, vitamins A, D, K, C, B6, B12, thiamin, riboflavin, niacin, pantothenic acid, biotin, folate, calcium, phosphorus, iron, zinc, copper, manganese, selenium, magnesium (P < 0.001), white meat (P = 0.001), and legumes (P = 0.023) were significant across quartiles of CSI, also a significant mean difference was observed among quartiles of the N6/N3 in terms of red meat, protein, carbohydrate, total fat, vitamins A, D, E, thiamin, riboflavin, niacin, pantothenic acid, B6, biotin, folate, B12, calcium, phosphorus, iron, zinc, copper, manganese, selenium, magnesium (P < 0.001), vegetables (P = 0.009), legumes (P = 0.015), and vitamin K (P = 0.043).

**Table 3:** Dietary intake of study population according to quartiles of CSI (n = 378) in obese and overweight women.

Variables	CSI				
	Mean ± SD				P-value*
	Q1 (n = 94)	Q2 (n = 95)	Q3 (n = 95)	Q4 (n = 94)	
Food group					
Grains (g/d)	205.51 ± 78.92	189.14 ± 63.76	183.11 ± 59.90	159.66 ± 49.12	** < 0.001**
Legumes (g/d)	19.65 ± 15.45	21.85 ± 17.99	18.80 ± 14.54	15.05 ± 13.04	**0.023**
Vegetables (g/d)	147.63 ± 85.10	168.94 ± 108.26	174.26 ± 97.51	159.14 ± 96.08	0.250
Fruits (g/d)	190.87 ± 122.97	189.42 ± 115.01	199.45 ± 103.43	172.88 ± 103.53	0.424
Dairy (ml/d)	116.61 ± 68.33	145.15 ± 80.76	156.71 ± 93.89	166.72 ± 86.93	** < 0.001**
Fast food (g/d)	7.71 ± 10.59	6.89 ± 5.71	8.44 ± 8.65	9.88 ± 8.67	0.105
White meat (g/d)	13.76 ± 9.75	17.33 ± 12.23	19.14 ± 14.53	22.27 ± 19.38	**0.001**
Red meat (g/d)	12.55 ± 8.69	15.16 ± 12.13	25.61 ± 20.53	33.61 ± 27.91	** < 0.001**
Nutrient intake					
Energy (kcal/d)	2116.76 ± 717.62	2365.63 ± 628.13	2825.02 ± 683.34	3197.17 ± 748.94	–
Protein (g/d)	66.54 ± 19.58	78.63 ± 19.76	100.22 ± 21.80	120.03 ± 33.11	** < 0.001**
Carbohydrate (g/d)	306.93 ± 116.35	340.04 ± 107.96	408.75 ± 119.37	430.84 ± 112.75	** < 0.001**
Total fat (g/d)	76.09 ± 31.14	84.73 ± 29.86	97.73 ± 26.02	120.19 ± 34.44	** < 0.001**
Vitamin A (mg/d)	547.56 ± 311.31	644.62 ± 286.31	858.66 ± 429.92	1003.49 ± 424.15	** < 0.001**
Vitamin D (ug/d)	1.11 ± 0.87	1.65 ± 1.18	2.12 ± 1.51	3.00 ± 1.90	** < 0.001**
Vitamin E (mg/d)	16.02 ± 10.45	16.67 ± 9.89	16.73 ± 6.66	18.33 ± 8.33	0.338
Vitamin K (mg/d)	212.20 ± 277.72	236.87 ± 250.19	318.97 ± 295.51	391.65 ± 326.26	** < 0.001**
Thiamin (mg/d)	1.76 ± 0.69	1.92 ± 0.62	2.30 ± 0.60	2.54 ± 0.75	** < 0.001**
Riboflavin (mg/d)	1.69 ± 0.73	1.93 ± 0.55	2.44 ± 0.59	3.05 ± 0.89	** < 0.001**
Niacin (mg/d)	20.11 ± 6.95	22.98 ± 6.82	28.23 ± 7.59	34.01 ± 12.19	** < 0.001**
Pantothenic acid (mg/d)	4.78 ± 1.51	5.70 ± 1.60	7.08 ± 1.91	8.24 ± 2.79	** < 0.001**
Vitamin B6 (mg/d)	1.65 ± 0.52	1.95 ± 0.59	2.42 ± 0.62	2.75 ± 0.77	** < 0.001**
Biotin (mg/d)	27.92 ± 12.13	33.66 ± 12.38	42.23 ± 13.73	49.51 ± 19.76	** < 0.001**
Folate (mcg/d)	580.44 ± 204.68	648.88 ± 223.05	744.87 ± 223.85	802.45 ± 229.64	** < 0.001**
Vitamin B12 (mcg/d)	2.69 ± 1.13	3.53 ± 1.57	4.61 ± 1.64	6.60 ± 2.21	** < 0.001**
Vitamin C (mg/d)	152.89 ± 135.61	163.41 ± 100.84	213.64 ± 105.58	222.86 ± 103.19	** < 0.001**
Calcium (mg/d)	938.01 ± 405.77	1098.33 ± 396.17	1359.52 ± 376.73	1698.72 ± 615.85	** < 0.001**
Phosphorus (mEq/d)	1251.75 ± 426.39	1457.46 ± 401.67	1848.07 ± 412.25	2144.39 ± 552.29	** < 0.001**
Iron (mg/d)	20.91 ± 20.88	22.57 ± 17.99	25.34 ± 13.68	37.84 ± 26.21	** < 0.001**
Zinc (mg/d)	10.02 ± 3.63	11.50 ± 3.44	14.92 ± 4.01	17.27 ± 4.81	** < 0.001**
Copper (mg/d)	1.62 ± 0.60	1.80 ± 0.61	2.22 ± 0.65	2.44 ± 0.83	** < 0.001**
Manganese (mg/d)	6.94 ± 4.08	7.06 ± 3.23	8.49 ± 3.44	9.70 ± 4.84	** < 0.001**
Selenium (mcg/d)	102.06 ± 47.06	110.36 ± 37.07	136.04 ± 38.38	156.63 ± 54.73	** < 0.001**
Magnesium (mg/d)	375.94 ± 155.79	418.77 ± 145.26	535.29 ± 148.12	573.59 ± 161.50	** < 0.001**

CSI: Cholesterol to saturated fat index; Q: quartile.

Data are mean ± SD.

P-value*: ANCOVA was performed to adjust the potential confounding factor (energy intake).

p < 0.05 was considered significant, and 0.05, 0.06, and 0.07 were considered marginally significant, and are bolded.

**Table 4:** Dietary intake of study population according to quartiles of N6/N3 (n = 279) in obese and overweight women.

Variables†	N6/N3				
	Mean ± SD				P-value*
	Q1 (n = 69)	Q2 (n = 70)	Q3 (n = 70)	Q4 (n = 70)	
Food group					
Grains (g/d)	179.50 ± 69.94	181.99 ± 60.01	181.73 ± 84.56	191.13 ± 58.87	0.762
Legumes (g/d)	14.89 ± 11.36	20.64 ± 17.87	23.31 ± 17.30	19.32 ± 14.68	**0.015**
Vegetables (g/d)	141.17 ± 84.52	156.78 ± 86.78	194.01 ± 114.32	177.26 ± 99.16	**0.009**
Fruits (g/d)	227.09 ± 106.73	199.77 ± 109.97	206.30 ± 111.13	204.79 ± 135.33	0.527
Dairy (ml/d)	141.60 ± 79.98	150.10 ± 79.31	150.22 ± 87.64	151.32 ± 80.01	0.890
Fast food (g/d)	10.19 ± 8.98	7.93 ± 8.10	7.35 ± 7.41	8.53 ± 11.71	0.300
White meat (g/d)	17.74 ± 18.97	15.97 ± 12.00	17.90 ± 14.26	21.24 ± 14.58	0.222
Red meat (g/d)	31.55 ± 19.21	26.68 ± 21.59	15.82 ± 15.29	12.83 ± 8.86	** < 0.001**
Nutrient intake					
Energy (kcal/d)	3633.72 ± 333.83	2806.24 ± 166.18	2296.03 ± 153.21	1697.45 ± 232.04	–
Protein (g/d)	119.71 ± 24.87	95.01 ± 17.89	79.41 ± 15.29	59.46 ± 12.84	** < 0.001**
Carbohydrate (g/d)	525.24 ± 80.79	395.54 ± 59.55	325.81 ± 35.81	239.38 ± 47.96	** < 0.001**
Total fat (g/d)	129.15 ± 26.56	102.98 ± 22.86	83.07 ± 15.49	60.66 ± 14.56	** < 0.001**
Vitamin A (mg/d)	1036.87 ± 444.98	755.59 ± 341.67	737.81 ± 419.19	555.73 ± 264.44	** < 0.001**
Vitamin D (ug/d)	2.63 ± 2.24	2.10 ± 1.47	1.76 ± 1.32	1.37 ± 0.98	** < 0.001**
Vitamin E (mg/d)	20.51 ± 9.18	19.11 ± 9.32	16.82 ± 9.34	12.50 ± 6.61	** < 0.001**
Vitamin K (mg/d)	246.58 ± 171.66	205.49 ± 137.57	234.61 ± 299.33	160.71 ± 99.24	**0.043**
Thiamin (mg/d)	2.80 ± 0.51	2.24 ± 0.36	1.86 ± 0.35	1.38 ± 0.28	** < 0.001**
Riboflavin (mg/d)	3.02 ± 0.84	2.32 ± 0.55	1.96 ± 0.51	1.47 ± 0.35	** < 0.001**
Niacin (mg/d)	35.01 ± 10.05	26.20 ± 5.22	22.35 ± 4.44	17.08 ± 4.11	** < 0.001**
Pantothenic acid (mg/d)	8.71 ± 2.85	6.90 ± 1.64	5.86 ± 1.20	4.39 ± 0.99	** < 0.001**
Vitamin B6 (mg/d)	2.91 ± 0.60	2.26 ± 0.47	1.98 ± 0.46	1.45 ± 0.31	** < 0.001**
Biotin (mg/d)	49.59 ± 21.36	41.39 ± 13.13	36.06 ± 12.55	26.00 ± 8.60	** < 0.001**
Folate (mcg/d)	903.42 ± 181.93	710.78 ± 164.04	610.88 ± 128.75	477.07 ± 124.12	** < 0.001**
Vitamin B12 (mcg/d)	6.16 ± 3.30	4.50 ± 1.87	3.64 ± 1.46	3.12 ± 1.39	** < 0.001**
Vitamin C (mg/d)	282.52 ± 153.97	196.14 ± 104.93	175.71 ± 98.88	125.17 ± 73.42	** < 0.001**
Calcium (mg/d)	1537.34 ± 370.69	1257.46 ± 352.77	1069.00 ± 309.15	787.21 ± 222.96	** < 0.001**
Potassium (mEq/d)	2174.36 ± 386.87	1782.10 ± 373.34	1480.13 ± 309.93	1086.65 ± 240.72	** < 0.001**
Iron (mg/d)	25.76 ± 4.09	19.83 ± 3.37	16.77 ± 3.00	12.10 ± 2.21	** < 0.001**
Zinc (mg/d)	17.48 ± 2.98	14.17 ± 2.72	11.57 ± 2.47	8.29 ± 1.64	** < 0.001**
Copper (mg/d)	2.75 ± 0.72	2.11 ± 0.41	1.79 ± 0.40	1.30 ± 0.29	** < 0.001**
Manganese (mg/d)	9.03 ± 2.61	7.75 ± 1.95	6.27 ± 1.73	4.96 ± 2.90	** < 0.001**
Selenium (mcg/d)	159.05 ± 39.67	131.21 ± 30.03	106.54 ± 28.37	79.63 ± 18.67	** < 0.001**
Magnesium (mg/d)	607.79 ± 106.04	498.08 ± 99.07	422.31 ± 95.21	299.08 ± 79.53	** < 0.001**

Q: quartile.

Data are mean ± SD.

P-value*: ANCOVA was performed to adjust the potential confounding factor (energy intake).

p < 0.05 was considered significant, and 0.05, 0.06, and 0.07 were considered marginally significant, and are bolded.

### Psychological disorders of study participants according to quartiles of CSI and N6/N3

The psychological disorders of study participants according to quartiles of CSI and N6/N3 were presented in Table 5. As shown in this table, in the crude model, and also after adjusting with confounders (age, energy intake, BMI, thyroid disease, and physical activity) the mean differences of stress, anxiety, depression, DASS-21 across quartiles of CSI and N6/N3 were not significant (P > 0.05).

**Table 5:** Psychological disorders of study participants according to quartiles of CSI (n = 378) and N6/N3 (n = 279) in obese and overweight women.

Variables	CSI				P-value†	P-value _b_
	Mean ± SD					
	Q1 (n = 94)	Q2 (n = 95)	Q3 (n = 95)	Q4 (n = 94)		
DASS-21	18.92 ± 12.66	18.64 ± 12.80	17.36 ± 9.70	17.72 ± 11.25	0.854	0.927
Depression	5.64 ± 4.97	5.60 ± 5.15	4.61 ± 3.65	4.92 ± 4.67	0.498	0.994
Anxiety	4.96 ± 4.26	5.05 ± 3.75	5.02 ± 3.64	5.13 ± 3.79	0.997	0.665
Stress	8.30 ± 5.18	7.98 ± 5.65	7.72 ± 4.39	7.66 ± 4.62	0.888	0.926

Variables†	N6/N3				P-value*	P-value _b_
	Mean ± SD					
	Q1 (n = 69)	Q2 (n = 70)	Q3 (n = 70)	Q4 (n = 70)		
DASS-21	16.78 ± 10.05	17.52 ± 10.79	19.18 ± 13.43	19.25 ± 12.10	0.555	0.703
Depression	4.72 ± 4.34	4.69 ± 4.01	5.83 ± 5.19	5.62 ± 4.97	0.363	0.673
Anxiety	4.47 ± 3.23	4.77 ± 3.73	5.15 ± 4.40	5.77 ± 3.87	0.270	0.679
Stress	7.59 ± 4.46	8.05 ± 4.96	8.19 ± 5.64	7.85 ± 4.90	0.914	0.676

CSI: Cholesterol to saturated fat index; Q: quartile; SD: standard deviation.

^†^ Calculated by analysis of variance (ANOVA).

b: Adjusted for age, BMI, physical activity, thyroid disease, and total energy intake.

p < 0.05 was considered significant, and 0.05, 0.06, and 0.07 were considered marginally significant.

### The interaction between genes related to lipid homeostasis with CSI and N6/N3 on psychological disorders in obese and overweight women

The interaction between genes related to lipid homeostasis with tertiles of the CSI and N6/N3 ratio on psychological disorders were presented in Tables 6 and 7. In the crude model, a positive interaction was observed between TC genotype of MC4R and CSI on depression (β = 0.30, CI = 0.04, 0.56, P = 0.023), and DASS-21 (β = 0.62, CI = − 0.32, 1.27, P = 0.062). After adjusting for age, energy intake, thyroid disease, physical activity, and BMI in model 1, the interaction between TC genotype of MC4R and CSI on depression (β = 0.39, CI = 0.12, 0.66, P = 0.004), and DASS-21 (β = 0.074, CI = 0.04, 1.44, P = 0.036) remained positive. In particular, TC-alleles carriers were characterized by higher depression and DASS-21 when had the highest following CSI, compared to CC homozygote. In addition, there were some marginal significant interactions between AG genotype of CAV-1 and N6/N3 ratio on depression in both crude (β = 13.44, CI = –0.99, 27.88, P = 0.068) and adjustment model1 (β = 16.83, CI = − 0.19, 33.85, P = 0.053) which shows higher adherence to N6/N3 ratio, with higher depression in AG-alleles carriers compared to GG homozygote.

**Table 6:** The interaction between MC4R with CSI (n = 378) and N6/N3 (n = 279) on psychological disorders in obese and overweight women.

Variable	CSI							N6/N3					
	MC4R	Crude			Model 1			Crude			Model 1		
	Allele	B	CI	P	B	CI	P	B	CI	P	B	CI	P
Stress	TT	0.08	− 0.19, 0.36	0.552	0.06	− 0.22, 0.35	0.681	− 8.91	− 22.51, 4.68	0.199	− 2.15	− 17.24, 12.93	0.779
	TC	0.14	− 0.13, 0.42	0.312	0.14	− 0.16, 0.45	0.373	− 12.87	− 27.81, 2.06	0.091	− 7.09	− 23.71, 9.52	0.403
	CC	Reference			Reference			Reference			Reference		
Anxiety	TT	0.01	− 0.19, 0.23	0.865	0.01	− 0.19, 0.23	0.877	− 2.02	− 12.44, 8.38	0.703	1.97	− 9.44, 13.38	0.108
	TC	0.17	− 0.04, 0.38	0.112	0.21	− 0.01, 0.44	0.073	− 10.36	− 21.80, 1.07	0.076	− 8.00	− 20.57, 4.56	0.212
	CC	Reference			Reference			Reference			Reference		
Depression	TT	0.16	− 0.09, 0.42	0.207	0.21	− 0.03, 0.47	0.097	− 8.43	− 21.07, 4.20	0.191	− 6.04	− 19.51, 7.43	0.379
	TC	0.30	0.04, 0.56	**0.023**	0.39	0.12, 0.66	**0.004**	− 13.77	− 27.65, 0.10	**0.052**	− 13.64	− 28.48, 1.19	0.072
	CC	Reference			Reference			Reference			Reference		
Dass-21	TT	0.27	− 0.38, 0.92	0.415	0.29	− 0.36, 0.94	0.381	− 19.37	− 51.00, 12.26	0.23	− 6.23	− 40.68, 28.22	0.723
	TC	0.62	− 0.32, 1.27	**0.062**	0.74	0.04, 1.44	**0.036**	− 37.01	− 71.75, − 2.26	**0.037**	− 28.74	− 66.68, 9.19	0.138
	CC	Reference			Reference			Reference			Reference		

CI: confidence interval; CSI: cholesterol to saturated fat index; Q: quartile.

GLM was performed to identify the interaction between genes related to lipid homeostasis with CSI and N6/N3 on psychological disorders.

Model 1 = adjusted for potential confounding factors including (age, energy intake, physical activity, thyroid disease, and BMI).

p < 0.05 was considered significant, and 0.05, 0.06, and 0.07 were considered marginally significant, and are bolded.

**Table 7:** The interaction between CAV-1 with CSI (n = 378) and N6/N3 (n = 279) on psychological disorders in obese and overweight women.

Variable	CSI							N6/N3					
	CAV-1	Crude			Model 1			Crude			Model 1		
	Allele	B	CI	P	B	CI	P	B	CI	P	B	CI	P
Stress	AA	− 0.01	− 0.28, 0.25	0.907	− 0.01	− 0.28, 0.25	0.918	1.79	− 11.98, 15.56	0.799	− 5.55	− 20.64, 9.52	0.470
	AG	− 0.08	− 0.39, 0.23	0.611	0.07	− 0.26, 0.41	0.683	10.91	− 4.58, 26.40	0.168	6.47	− 12.43, 25.37	0.502
	GG	Reference			Reference			Reference			Reference		
Anxiety	AA	− 0.08	− 0.29, 0.12	0.418	− 0.11	− 0.32, 0.08	0.260	5.40	− 5.17, 15.98	0.316	6.19	− 5.33, 17.71	0.292
	AG	− 0.09	− 0.32, 0.15	0.463	0.02	− 0.23, 0.27	0.869	3.49	− 8.404	0.565	2.26	− 12.17, 16.70	0.758
	GG	Reference			Reference			Reference			Reference		
Depression	AA	− 0.12	− 0.36, 0.13	0.346	− 0.12	− 0.37, 0.11	0.307	4.06	− 8.77, 16.89	0.535	4.14	− 9.43, 17.73	0.550
	AG	− 0.28	− 0.57, 0.009	**0.057**	− 0.17	− 0.48, 0.12	0.251	13.44	− 0.99, 27.88	**0.068**	16.83	− 0.19, 33.85	**0.053**
	GG	Reference			Reference			Reference			Reference		
Dass-21	AA	− 0.22	− 0.84, 0.40	0.488	− 0.25	− 0.87, 0.36	0.412	11.26	− 20.80, 43.33	0.491	4.78	− 29.78, 39.30	0.786
	AG	− 0.45	− 1.17, 0.27	0.223	− 0.08	− 0.85, 0.68	0.828	27.85	− 8.22, 63.93	0.130	25.57	− 17.70, 68.85	0.247
	GG	Reference			Reference			Reference			Reference		

CI: confidence interval; Q: quartile.

GLM was performed to identify the interaction between genes related to lipid homeostasis with CSI and N6/N3 on psychological disorders.

Model 1 = adjusted for potential confounding factors including (age, energy intake, physical activity, thyroid disease, and BMI).

p < 0.05 was considered significant, and 0.05, 0.06, and 0.07 were considered marginally significant, and are bolded.

## Discussion

To the best of our knowledge, the present study is the first cross-sectional study to investigate the interaction between fatty acid quality indices and genes related to lipid homeostasis on stress, anxiety and depression among overweight and obese women. Accordingly, our results showed that there was an interaction between MC4R, CAV-1 genotypes and dietary fat quality indexes (CSI, W6/W3 ratio) on psychological disorders in overweight and obese women.

Our findings showed that a positive interaction between TC allele carriers of MC4R and higher adherence of CSI on depression and DASS-21. Considering that no studies have been conducted directly in this field, the results were analyzed based on some related studies that examined the components of our study. Yilmaz et al., in a study conducted in European adults, reported that MC4R was associated with increased depressive mood^25^. Also, in another study, it was shown that there was a significant positive interaction between the MC4R minor allele genotype, who also had higher fat intake, with mental stress in Korean adults^26^. This gene is associated with increased appetite (especially towards fats) and decreased satiety with the risk of weight gain^27^. Studies have shown that factors related to obesity, including increased body fat and intake of saturated fatty acids, and finally, inappropriate eating habits are associated with an increase in inflammatory markers, which can lead to an increased risk of depression^28–30^. Some animal evidence via the hypothalamicpituitary‐adrenal (HPA) axis have shown the relationship between MC4R and mental stress and depression and food intake^31,32^. They also stated that disruption of MC4R neutralizes the effects of antidepressants^33^.

According to our results, increased adherence to N6/N3 ratio in the interaction with CAV1 genotype (AG-alleles carriers) leads to a positive interaction on depression. Animal pharmacological studies have shown that CAV1 gene expression changes are associated with depression-like behaviors^34^. CAV1 is considered as one of the main components of Caveolae membrane and a group of integrated membrane proteins and inflammatory stimuli can increase the expression of the CAV1 gene, which itself leads to the intensification of inflammatory signaling events^35^. It has been reported that a high-fat diet is associated with increased CAV1 secretion from adipose tissue in mice^36^. In addition, high consumption of SFA can be associated with adverse effects of CAV1 in increasing the risk of metabolic syndrome and obesity^37^. Obesity is considered an inflammatory condition that can affect the state of the brain in a way that predisposes a person to depression^4^. Studies have shown that a higher ratio of N6/N3 PUFAs is associated with an increased risk of depressive symptoms^38,39^. The possible mechanism of this relationship is related to inflammatory responses, such that a high ratio of N6/N3 PUFAs increases the production of arachidonic acid derived from N6, which in turn is associated with an increase in pro-inflammatory factors and may cause Increase depression^40,41^.

The present study has several limitations. This study is cross-sectional and prevents inference of causality. In addition, the use of a self-report questionnaire that depended on memory and was prone to bias. This study was conducted with a small sample size only on the women population, so it could not be generalized. Despite the mentioned limitations, our study has strong points such as being the first study that investigated the interaction between fatty acid quality indices and genes related to lipid homeostasis on stress, anxiety and depression among overweight and obese women. Also, trained people were used to collect data to minimize bias.

## Conclusion

Based on the findings of the study, we found that there was a positive relationship between increased adherence to CSI and depression in TC allele carriers of MC4R, and there was also a positive relationship between higher N6/N3 ratio and depression in AG-allele carriers of CAV1. However, more studies in different populations are needed to confirm the findings.

## Data Availability

The data that support the findings of this study are available from Khadijeh Mirzaei but restrictions apply to the availability of these data, which were used under license for the current study, and so are not publicly available. Data are however available from the authors upon reasonable request and with permission of Khadijeh Mirzaei (mirzaei_kh@tums.ac.ir).

## Abbreviations

BF%: Body fat percentage
BFM: Body fat mass
BMI: Body mass index
CAV-1: Caveolin-1
CIs: Confidence intervals
CSI: Cholesterol-saturated fat index
DASS-21: Depression Anxiety Stress Scales
FFM: Fat free mass
FFMI: Fat free mass index
FMI: Fat mass index
GLM: Generalized linear model
HDL-C: High density lipoprotein cholesterol
hs-CRP: High-sensitivity C-reactive protein
IPAQ: International physical activity questionnaire
LDL-C: Low density lipoprotein cholesterol
MC4R: Melanocortin-4 receptor
PA: Physical activity
PUFA: Polyunsaturated fatty acid
SDs: Standard deviations
SFA: Saturated fatty acid
SNP: Single nucleotide polymorphisms
SLM: Soft lean mass
SMM: Skeletal muscle mass
TC: Total cholesterol
TG: Triglyceride
VFL: Visceral fat level
WC: Waist circumference
WHR: Waist-hip ratio
